# Research on the Distribution Characteristics of Urea-Formaldehyde Resin Gel Influenced by Structural Development in Fractured-Vuggy Reservoirs

**DOI:** 10.3390/gels11110868

**Published:** 2025-10-30

**Authors:** Zhengcong Song, Weipeng Wu, Ming Qu, Jiaxin Xi, Min Yang, Xingliang Jia, Yuheng Zhao, Lu Liu, Haihua Cui

**Affiliations:** 1 Sinopec Northwest Oilfield Company, Urumqi 830014, China; songzhengc.xbsj@sinopec.com (Z.S.); yangm.xbsj@sinopec.com (M.Y.); jiaxl.xbsj@sinopec.com (X.J.); liul8340.xbsj@sinopec.com (L.L.); cuihh.xbsj@sinopec.com (H.C.); 2 Sanya Offshore Oil and Gas Research Institute, Northeast Petroleum University, Sanya 572025, China; m.qu@foxmail.com; 3 Oil and Gas Technology Research Institute of PetroChina Changqing Oilfield Company, Xi’an 710018, China; xijiaxin_cq@petrochina.com.cn; 4 Unconventional Petroleum Research Institute, China University of Petroleum, Beijing 102249, China; z13633831585@163.com

**Keywords:** core slice, fractured-vuggy model, online NMR analysis, URG, 3D printed technology

## Abstract

Profile control is widely employed to improve oil recovery in fractured-vuggy carbonate reservoirs. However, the limitation of current experimental evaluation methods restricts their practical guidance for field applications. In this study, urea-formaldehyde resin gel (URG) is studied using SEM, rheological analysis, FTIR, and Raman spectroscopy. Typical structural models of fractured-vuggy reservoirs are fabricated by 3D printing technology. The distribution patterns of the URG in different fractured-vuggy models are also investigated by using online NMR analysis and core slice characterization. Results show that URG exhibits a kind of 3D mesh structure with a size of 10 μm after gelation at 140 °C. The storage modulus (G′) and loss modulus (G″) of the URG gel are 387.51 Pa and 131.48 Pa, respectively. Chemical composition analysis reveals that URG is mainly composed of amide groups and sulfonate groups, showing excellent thermal stability and salt tolerance. Furthermore, after injecting URG into three types of typical models, URG displays a longitudinally decreasing distribution pattern from the injection side to the outlet side, accompanied by transverse accumulation phenomenon along the fracture walls in the slab fracture model. In the fractured-vuggy model, the gel shows continuous longitudinal distribution and uniform transverse distribution characteristics. In the beaded-vug train model, the gel’s distribution morphology gradually transforms from a “pipeline-filling” pattern at the injection side to a “conduit-dominant” pattern toward the outlet side, with a stepped distribution in the transverse direction. The breakthrough pressures during subsequent water flooding are as follows: beaded-vug train model (11.6 MPa) > fractured-vuggy model (8.1 MPa) > slab fracture model (5.9 MPa). Field application results show that the water cut is reduced from 85% to 30%, with a total incremental oil production of 2416 tons. This study conducts experimental investigations on the distribution patterns of URG in simulated fractured-vuggy models, thereby establishing a novel technical evaluation method for profile control in actual fractured-vuggy carbonate reservoirs.

## 1. Introduction

Carbonate reservoirs form approximately two thirds of global proven hydrocarbon reserves. Fractured-vuggy reservoirs in particular account for more than 30% of the total volume [1,2]. Tahe Oilfield, China, is a quintessential Ordovician fractured-vuggy carbonate reservoir whose proven reserves have exceeded 1.41 billion tons [3]. However, these reservoirs exhibit unpredictable pore space distribution, substantial variations in reservoir body dimensions, and pronounced heterogeneity, leading to intricate fluid flow behavior [4,5]. Furthermore, current recovery efficiency is less than 15% which is ascribed to an average burial depth exceeding 5000 m, a geothermal gradient of 2.2 °C/100 m, formation water salinity generally above 200,000 mg/L, and calcium–magnesium ion concentrations exceeding 10,000 mg/L [6,7,8]. After years of water flooding and high-energy edge/bottom water influx, serious preferential flow channels have developed, leaving significant quantities of remaining oil effectively untapped [6]. Consequently, water control in fractured-vuggy reservoirs is far more complex than in sandstone reservoirs.

Over the past decade, fractured-vuggy carbonate reservoirs in Tahe oilfields have entered a development bottleneck state. Due to strong reservoir heterogeneity inherent to fracture-vuggy reservoirs and the formation of water-flooding channels, high water-cut wells proportion continue to rise [9,10]. Therefore, various targeted measures have been implemented to effectively control water cut rise in fractured-vuggy carbonate reservoirs in Tahe oilfields. Among these, water shutoff technologies, including physical and chemical methods, account for 28% of the total efforts [11,12,13]. The physical methods mainly rely on the installation of packers or downhole oil-water separators in the wellbore, but it requires high demands on the wellbore structure and detailed description of the reservoir [14,15]. Chemical methods mainly involve injecting agents such as polymer gels, inorganic gels, foam agents, and microbial agents into the wellbore to selective plugging targets in the dominant water channels [16,17]. Considering economic efficiency and operational convenience, current water shutoff technologies have transformed from physical to chemical water shutoff techniques in fractured-vuggy carbonate reservoirs in Tahe oilfields.

At present, crosslinked polymer gels are mainly used in fractured-vuggy carbonate reservoirs in Tahe oilfields. The pre-mixed gel solution is pumped into the formation. During a period of time, it forms a high-strength gel that effectively blocks water channeling, thereby achieving the dual objectives of water control and enhanced oil recovery [18,19,20]. Many researchers mainly conducted plugging tests by using core or sand-packed models, analyzing plugging efficiency and oil recovery based on experimental data [21,22,23]. For example, Sina Afsharpour et al. demonstrated that a gel system containing 1 wt% n-SiO_2_ exhibited the highest loss factor by employing a dual-permeability model, making it particularly suitable for water shut-off applications due to its lower injection pressure [24]. Their study further emphasized the superior effectiveness of such treatments in formations with wider fractures [24]. Bai et al. investigated the propagation and filling behavior of LPND gelant in a 2D fracture model, revealing a positive correlation between injection volume and oil recovery [25]. Specifically, as the injected gelant volume increased from 0.20 PV to 0.60 PV, the incremental oil recovery after gel plugging increased from 43.55% to 53.97% OOIP [25]. Ma et al. found that bulk gel systems displayed a breakthrough pressure of 42.10 psi/ft at a flow rate of 0.75 cm^3^/min [26]. Fang et al. found that the density of plugging agent equal to that of injected water is the key factor to realize the in-depth migration [27]. Wu et al. proposed distinct enhanced oil recovery factors across different types of fracture-vuggy structures by conducting water plugging experiments using visualized physical simulation models [9]. However, current understanding of gel imbibition-transport coupling mechanisms in heterogeneous porous media remains significantly inadequate. Therefore, it is significant to study the gel’s plugging mechanisms in cores/models by using advanced analysis techniques [28].

In this study, authors designed a novel bulk gel system named URG, which is applicable to fractured-vuggy carbonate reservoirs with high temperature of 140 °C, salinity of 240,000 mg/L, and calcium–magnesium ion content exceeding 10,000 mg/L. Additionally, temperature-resistant and pressure-resistant 3D models with different structures were fabricated. During each of the models, the injection, plugging and distribution of URG are evaluated in fractured-vuggy simulation models based on core slabbed and NMR T_2_ spectrum analysis. The authors clarify the plugging mechanisms of conformance control agents in fractured-vuggy structures and reveal their distribution patterns using core NMR methods. The proposed method provides innovative technical methodologies and theoretical foundations to improve water conformance control efficiency.

## 2. Results and Discussion

### 2.1. Morphologies and Physicochemical Properties of URG

**Figure 1:**
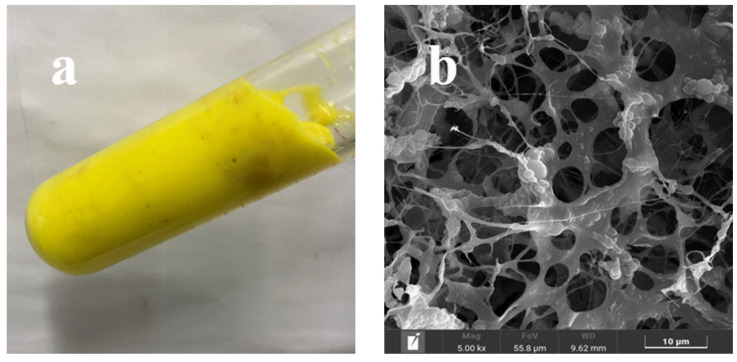
Morphologies of URG: (**a**) gelated, (**b**) SEM image.

The synthesized URG bulk gel is shown in Figure 1a. The pre-processed solution is transferred into an oven and heated for 5–6 h at 140 °C to form bulk gel. The SEM image of the microstructure of URG is shown in Figure 1b. The cross-linking between amide groups (−CONH_2_) and hydroxymethyl groups generates hydroxymethylated amide-based branched chains. This process inhibits thermal hydrolysis of amide groups, reduces the thermal degradation rate of the polymer and contracts the 3D mesh structure of the gel. Ultimately, an irregular layered stacking morphology with a minimum pore size of approximately 10 μm is formed. Therefore, URG demonstrates excellent thermal stability and superior mechanical strength.

**Figure 2:**
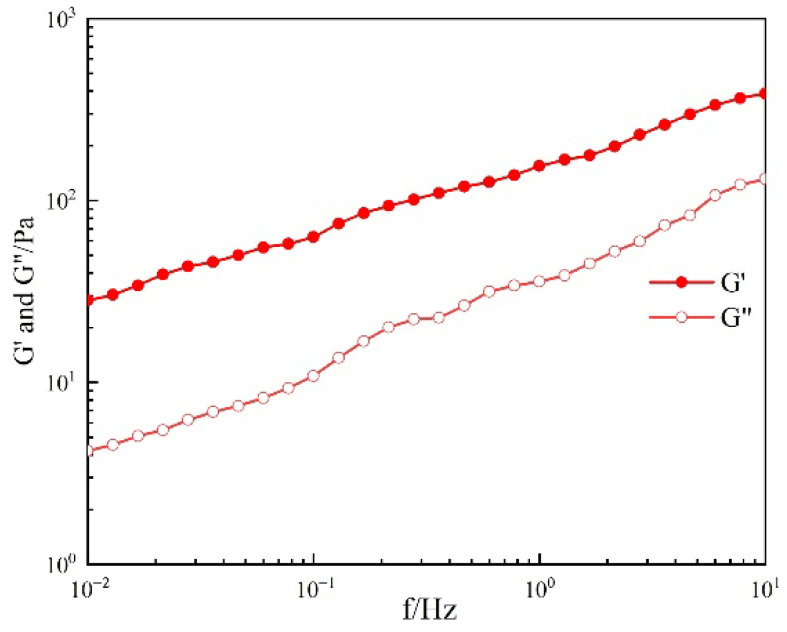
Storage modulus (G′) and loss modulus (G″) of URG after gelated.

The URG bulk gel system after being gelated completely is primarily governed by its energy storage modulus (G′) and loss modulus (G″), which respectively reflect the crosslinking efficiency and viscoelastic behavior under operational conditions. Commonly, G′ represents the material’s ability to store elastic deformation energy, reflecting the structural strength of the cross-linked network. A higher G′ indicates greater resistance to impact and local damage after deforming. G″ represents the material’s ability to dissipate energy as heat during deformation, reflecting its viscous energy loss characteristics. The lower the G″, the weaker the internal friction resistance and erosion resistance. As shown in Figure 2, G′ and G″ of URG after being gelated are 387.51 Pa and 131.48 Pa, respectively. Here, hydrogen bonds as physical crosslinking points enhance the network’s stiffness. The dynamic dissociation–recombination processes of hydrogen bonds contribute to energy dissipation. Concurrent enhancement of storage modulus (G′) and loss modulus (G″) indicates a coupled reinforcement of elastic and viscous properties in the material network. URG governed by hydrogen bonds exhibit predominantly elastic behavior (tan δ = G″/G′ = 0.33) and shear-thinning characteristic [29].

**Figure 3:**
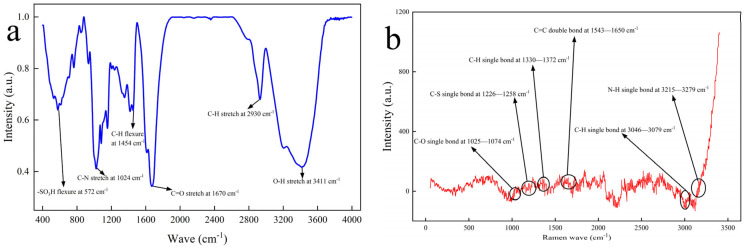
Functional group characterization of URG: (**a**) FT-IR spectrum, (**b**) Raman spectrum.

The functional groups of URG are characterized using FT–IR (Agilent, San Clara, CA, USA) and Raman Imaging Microscope (ThermoFisher, Waltham, MA, USA), as shown in Figure 3. As seen Figure 3a, URG has a characteristic peak at 3949 cm^−1^, which is attributed to the O–H stretching vibration of free hydroxyl groups. Then, a characteristic peak at 2929 cm^−1^ is ascribed to the C–H stretching vibration of aliphatic series. Conjugation shifts the C–H out-of-plane bending vibration to 1464 cm^−1^ and C=O stretching band of ester groups appears at 1700 cm^−1^. In addition, the high intensity absorption peak at 1052 cm^−1^ is ascribed to C=O bond and the peak at 629 cm^−1^ is due to the bending vibrational -SO_3_H bond [30,31,32]. The Raman analysis diagram of URG is shown in Figure 3b. A distinct Raman shift at 1025–1074 cm^−1^ is attributed to the low-frequency C–O single bond vibrational mode. The characteristic peak at 1226–1258 cm^−1^ is attributed to the C–S single bond vibration of aliphatic series. The characteristic peak at 1330–1372 cm^−1^ is attributed to the high-frequency C–H single bond vibration. The characteristic peak at 1543–1650 cm^−1^ is attributed to conjugated C=C double bond stretching vibrations. The characteristic peak at 3046–3079 cm^−1^ is attributed to C–H single bond vibration of aromatic compounds. N–H single bond of amine group at 3215–3279 cm^−1^ stretches the vibrations peak [33,34,35]. The asymmetric stretching vibration exhibits higher intensity than the symmetric stretching mode. The high-temperature stability and salt resistance can be improved because the URG is mainly composed of an amide group and sulfonate group.

**Figure 4:**
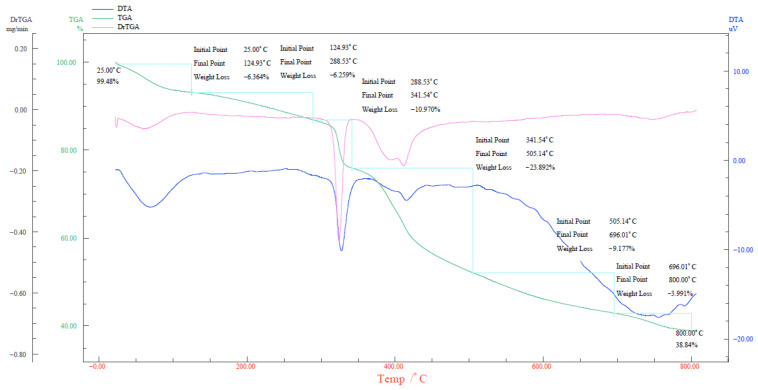
TG-DSC of URG.

Based on the TG–DSC analysis presented in Figure 4, the weight of URG begins to undergo glass transition below 125 °C, accompanied by a weight loss of 6.346%, which is attributed to the evaporation of free water. In the temperature range of 125–288 °C, the system enters an exothermic stage, leading to a weight loss of 6.259% due to the decomposition of uncross-linked polymers. As the temperature rises to 288–341 °C, the gel transforms into a melting stage. Within this stage, molecular chains of hydrophilic groups in the gel system break and dehydration effects continue to intensify, resulting in a weight loss of 10.97% caused by the disruption of the URG network structure. As temperatures exceed 341 °C, the system enters a state of decomposition and gasification. TG scanning results indicate that the continuous cleavage of the molecular backbone gradually slows down. Internally, the gel structure loses intermolecular association and attractive forces, leading to dehydration and degradation upon heating. Consequently, within the typical temperature range of oil reservoirs (25–341 °C), the cumulative weight loss is only 23.5%, demonstrating relatively excellent thermal stability.

### 2.2. Distribution Characteristics of URG in Fabricated Slab Fractures Core

**Figure 5:**
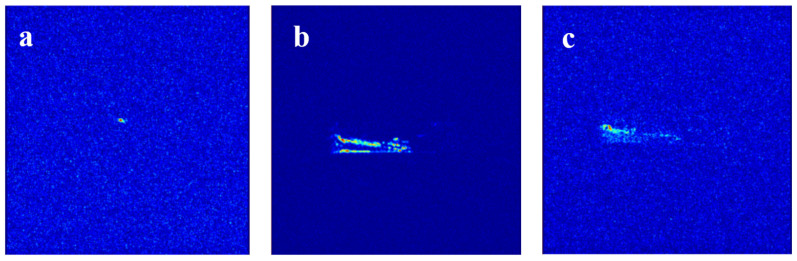
NMR sagittal images of slab fractures core: (**a**) Initial water flooding, (**b**) URG injection, (**c**) Subsequent water flooding.

The slab fractures core is dried and vacuumed, then placed into the experimental instrument. The online NMR plugging experiment is conducted following the aforementioned experimental steps. The processes are illustrated in Figure 5. Initially, when the core is fully saturated with deuterium oxide (D_2_O), none of the components are present within its internal pore structure. As shown in Figure 5a, no significant signal sources are detected in the corresponding NMR image, consistent with the pore space being occupied solely by D_2_O. Subsequently, 0.3 PV of URG solution is injected into the core and aged for 48 h until URG complete gelation. The NMR image in Figure 5b indicates a gradual attenuation of the signal from the inlet toward the outlet. This indicates that the distribution concentration of URG gradually decreases from the inlet toward the outlet affected by the internal structure of the slab fractures core. Additionally, due to the existence of minor flow channels at the distal end of the slab fractures core, the majority of the URG solution is uniformly distributed from the inlet to the middle section while the remaining portion gel exhibits lower concentration toward the outlet during migration. Finally, after 0.3 PV of URG complete gelation in the slab fractures core, it forms a pattern characterized by predominant accumulation at the inlet face and sporadic distribution in the outlet region. During subsequent water flooding, the NMR image is shown in Figure 5c, which indicates weaker signals from the areas of primary gel accumulation. The subsequent water induces bypass flow channeling in the slab fracture network.

**Figure 6:**
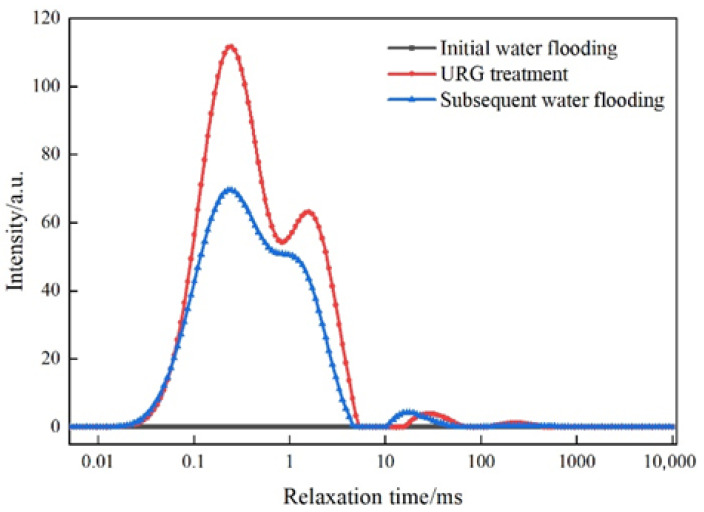
Dynamic NMR T_2_ curve of injection and plugging period of URG in slab fractures core.

The variations of NMR T_2_ spectrum curves at different flooding stages are shown in Figure 6. During initial water flooding, no significant changes in signal intensity peaks can be observed. Then, signal intensity peaks occur within the same relaxation time range during both the URG solution injection and the subsequent water flooding. Among them, when URG solution is injected, the concentrated and high-intensity relaxation time indicates a relatively uniform distribution of internal structures in the slab fractures core. Another signal peak shows no significant shift during subsequent water flooding, indicating that the URG has formed a high-strength blockage zone, resulting in the decrease of signal intensity. Based on the aforementioned trends, the authors suggest that URG can effectively reside in the internal space, forming a sealing zone from the inlet to the outlet with gradually decreasing intensity. The gelled state formed within this zone demonstrates effective sealing performance and strong erosion resistance in the internal structure of the slab fractures core [12,17].

**Figure 7:**
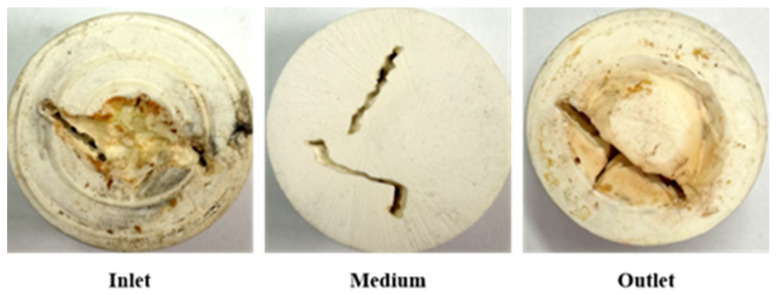
Distribution of URG in slab fractures core.

The core slice after URG is aged in the internal space of the slab fractures core is shown in Figure 7. The URG concentration gradually decreases longitudinally from the inlet to the outlet. The URG gel primarily accumulates along the wall surface at the inlet, forming a sealing layer with certain strength. During subsequent water flooding, the pressure rise is influenced by changes of flow paths. When the gel migrates toward the outlet in the fracture channel with the increase of the fracture aperture, the URG storage modulus changes. Under compression, the URG appropriately migrates toward wider fractures driven by the internal fracture width development of the model structure. When the fracture aperture gradually decreases, the migration capacity of URG declines. The gel becomes more susceptible to being breached by the continuous scouring of subsequent water flooding after URG complete gelation [36,37].

### 2.3. Distribution Characteristics of URG in Fabricated Fractured-Vuggy Core

**Figure 8:**
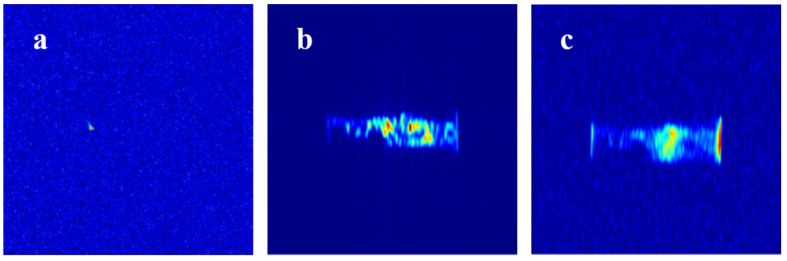
NMR sagittal images of fractured-vuggy core: (**a**) Initial water flooding, (**b**) URG injection, (**c**) Subsequent water flooding.

The procedure for the online NMR plugging experiment on fractured-vuggy core is consistent with that used for slab fractures core, as shown in Figure 8. When the slab fractures core is saturated with D_2_O, there are no discernible signal sources in the NMR image (Figure 8a). Then, 0.3 PV of URG solution is injected into the core and aged for 48 h until URG complete gelation. The NMR image in Figure 8b shows the internal pore space of the core sample. Due to the integrity of the continuous gel system in URG, there is no gel slug in the micro-flow channels at the inlet of the fractured-vuggy core. However, owing to the non-Newtonian fluid characteristics of URG, it continuously migrates toward the mid-distal region of the fractured-vuggy core with uniform dispersion. Influenced by the discontinuity of the internal spatial development structure in the fractured-vuggy core, URG fills all effective spaces within the core in a block-like distribution pattern. Therefore, URG predominantly exhibits continuous and uniform distribution in the fabricated fractured-vuggy core. Based on this observed phenomenon, subsequent water flooding is conducted. The corresponding NMR image (Figure 8c) demonstrates that URG undergoes progressive compression and aggregation, migrating from small-scale fractures in the central region toward large-scale vugs. This process enhances both the plugging strength and erosion resistance of URG.

**Figure 9:**
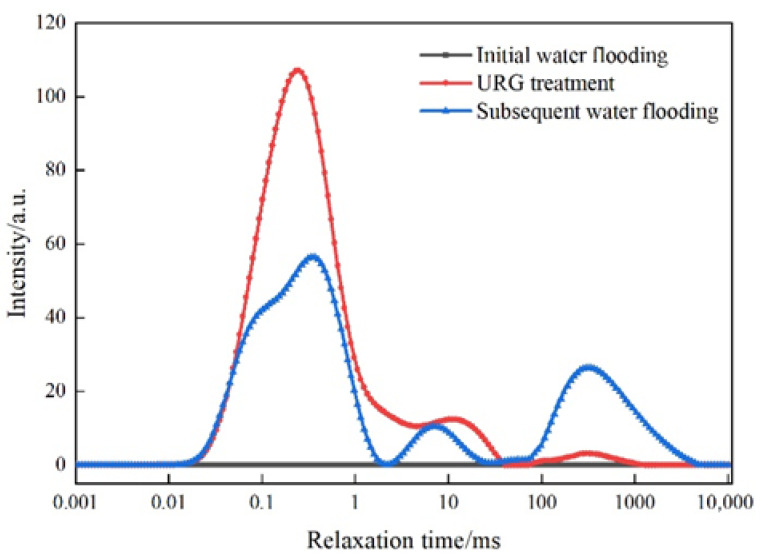
Dynamic NMR T_2_ curve of injection and plugging period of URG in fractured-vuggy core.

The variations of NMR T_2_ spectrum curves at different flooding stages are curved in Figure 9. During initial water flooding, no significant changes in signal intensity peaks can be observed owing to the presence of well-developed flow pathways and porous spaces in the fractured-vuggy core. Then, the NMR T_2_ spectrum curve exhibits prolonged relaxation time and significant intensity fluctuations after URG solution injection, indicating substantial variations in pore-size distribution within the internal structure of the fracture-cavity core. URG exhibits varying degrees of retention across multiple spatial domains, while maintaining generally uniform overall distribution. Based on the aforementioned trends, the significant rightward shift of the signal peak and the emergence of new peaks confirm the effective post-gelation plugging performance of URG within the fractured-buggy core. Even under continuous erosion from subsequent water flooding, the loosely distributed URG migrates toward large-scale deep spaces, while maintaining structural integrity within the highly heterogeneous fractured-buggy core. This process enhances the effective utilization rate of URG and reinforces both the stability and strength of the gel slug [12,17].

**Figure 10:**
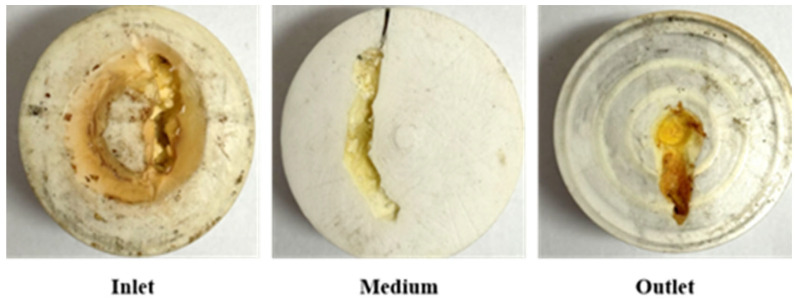
Distribution of URG in fractured-vuggy core.

The core slice after URG aged in the internal space of the fractured-vuggy core is shown in Figure 10. URG exhibits uniform distribution with continuous spatial continuity at the inlet interface of the fractured-vuggy core. As the flow pathway progresses toward the outlet, the internal flow space of URG constricts, occasionally leading to localized accumulation at zones with limited connectivity. During subsequent water flooding, portions of the URG system with weaker wall contact gradually migrate and accumulate into larger internal spaces, forming high strength sealing segments. Therefore, the increased breakthrough pressure of the URG bulk phase promotes its gradual secondary accumulation within large-scale spaces in the fractured-vuggy core structure, consequently enhancing the overall structural stability [36,37].

### 2.4. Distribution Characteristics of URG in Fabricated Beaded-Vug Train Core

**Figure 11:**
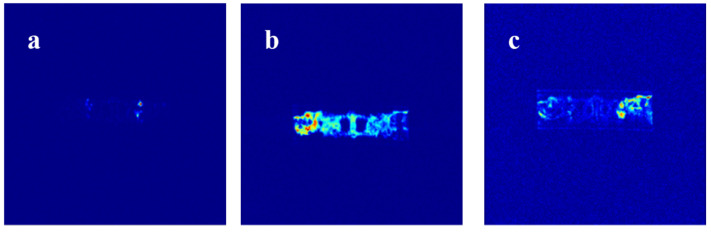
NMR sagittal images of beaded-vug train core: (**a**) Initial water flooding, (**b**) URG injection, (**c**) Subsequent water flooding.

The procedure for the online NMR plugging experiment on beaded-vug train core is shown in Figure 11. Similarly to the slab fracture core and fractured-vuggy core, no significant signal sources can be observed in the NMR image (Figure 11a) during initial water flooding through the chain-like structure’s internal channel. Then, 0.3 PV of URG solution is injected into the core and aged for 48 h until URG complete gelation. The NMR image (Figure 11b) shows that the beaded-vug train core contains multiple segments of discontinuous fine flow channels from the inlet to the outlet. Specifically, during URG migration from the inlet to outlet, URG achieves uniform distribution and accumulation exclusively in zones with well-maintained flow continuity and sufficient spatial dimensions, while forming clustered distributions in isolated small-aperture regions. Throughout the internal space of the beaded-vug train core, the URG retention concentration progressively decreases from the inlet to the outlet, exhibiting a step-like distribution pattern. Only minimal URG remains in the distal end of the beaded-vug train core because dominant water flow channels have become established. The corresponding NMR image (Figure 11c) indicates that a portion of URG remains in the beaded-vug train core as with heavy water flooding. While the remaining URG undergoes migration, the colloidal aggregates initially concentrated in the anteromedial section of the model gradually migrate into deeper secondary channels with progressive water injection. URG can result in the formation of plugging slugs because of the cementation capacity between URG and small-aperture core surfaces.

**Figure 12:**
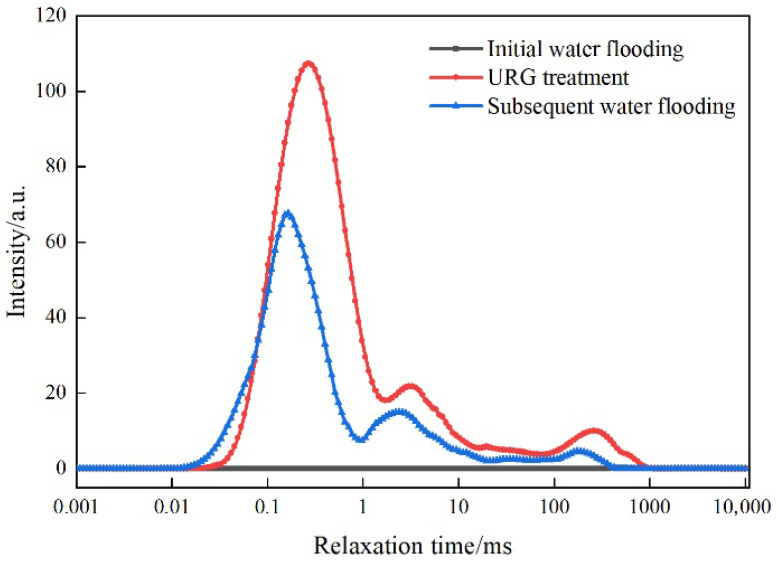
Dynamic NMR T_2_ curve of injection and plugging period of URG in beaded-vug train core.

The variations of NMR T_2_ spectrum curves at different flooding stages are shown in Figure 12. During initial water flooding, no significant changes in signal intensity peaks can be observed. Then, prolonged relaxation time accompanied by significant intensity fluctuations is observed when the URG solution is injected. Owing to the substantial variations in pore-size distribution within the internal structure of the beaded-vug train core, URG forms sealing layers across multiple large-scale apertures while maintaining considerable continuity. The overall leftward shift of signal indicates that URG gel migrates gradually into smaller flow channels. Therefore, URG achieves effective plugging throughout all stages in the beaded-vug train core [12,17].

**Figure 13:**
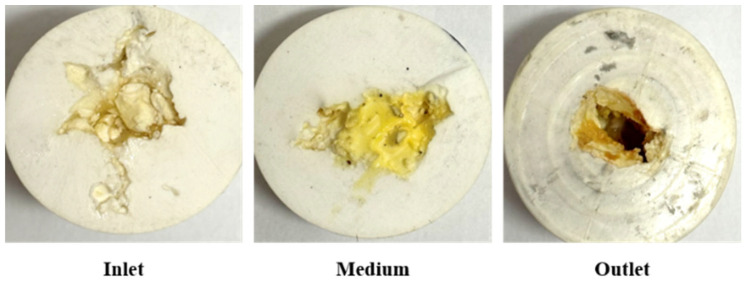
Distribution of URG in beaded-vug train core.

The core slice after URG aged in the internal space of the beaded-vug train core is shown in Figure 13. Within the beaded-vug train structure, URG achieves complete and dense packing at the inlet interface, while simultaneously distributing uniformly along multiple pathways toward the outlet following the irregular development of flow channels. Within major large-scale seepage channels, the URG concentration gradually decreases with concurrent coalescence along the walls, while the transverse migration process of URG is governed by variations in fracture aperture. Therefore, within the complex developmental spaces of the beaded-vug train core, URG sequentially undergoes transitions from an extended state to a compressed state, and then re-enters an extended state. The URG gel can maintain high viscoelasticity overall and also enhance plugging efficiency within internal flow channels.

### 2.5. Displacement Pressure Differential of URG in Different Fractured-Vuggy Structures

**Figure 14:**
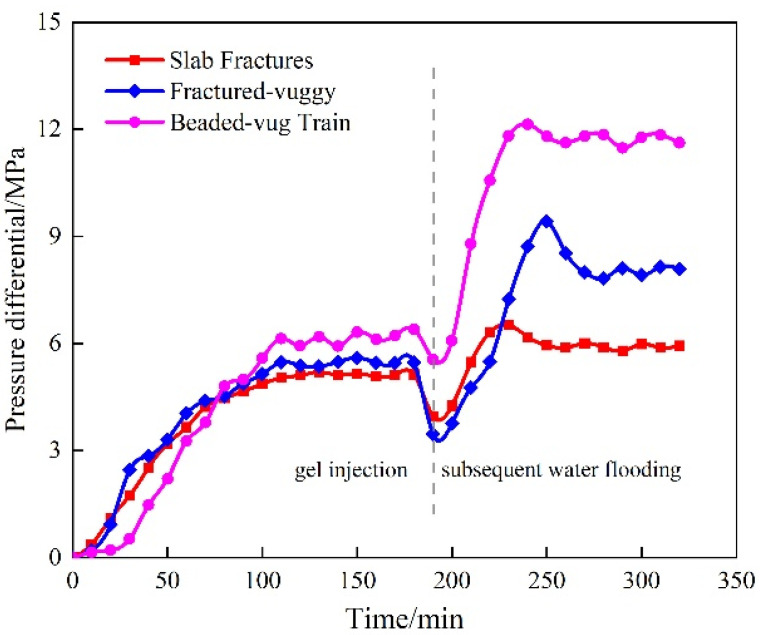
Displacement pressure differential of URG in different fractured-vuggy structures.

In this section, URG is injected into three representative 3D-printed fractured-vuggy core models. These models simulate the predominant flow pathways observed in actual reservoirs. The measured displacement pressure differentials are shown in Figure 14. All models are fabricated with uniform dimensions of ϕ25 mm × 100 mm. When URG solution is injected, the displacement pressure differential exhibits varying changes due to the influence of the internal spatial structure development of the models. In slab fractures core, the displacement pressure differential increases steadily during the injection process. The fractured-vuggy core has a small channel at the inlet, followed by the development of cavity space into fracture space. As a result, the displacement pressure differential increases significantly at the beginning of injection and then gradually keeps steady. In the beaded-vug train core, the inlet end develops predominantly with large vug morphology, followed by multiple vug connected in a bead-like pattern via fractures. The displacement pressure differential increases significantly in the intervals where fractures connect the vugs. Subsequent water flooding experiments are conducted after URG injection for 8–10 h. The displacement pressure differential increases rapidly, indicating that URG possesses a certain plugging ability in different fractured-vuggy reservoirs. However, the breakthrough pressure during subsequent water flooding varies significantly due to differences in the development of fractured-vuggy structures. The breakthrough pressure for subsequent water flooding in slab fractures core is 5.9 MPa, while the breakthrough pressure is 8.1 MPa in fractured-vuggy core and 11.6 MPa in beaded-vug train core. Therefore, based on the experimental results of breakthrough pressure and distribution characteristics of URG in different 3D-printed fractured-vuggy core structures, it can be concluded that URG gel exhibits excellent adaptability for implementing water shut-off in reservoir spaces developed with bead-like cavities, fracture-cavity systems, and slab-shaped fractures [36,37].

### 2.6. Applications in Fractured-Vuggy Reservoirs In-Situ

**Figure 15:**
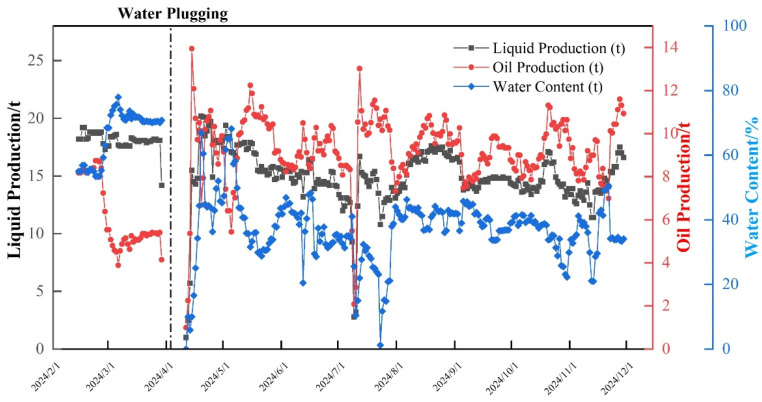
Production curve of THXX264 well before and after water plugging.

TKXX264 well in fractured-vuggy reservoir of Tahe Oilfield is a typical fault-controlled karst-developed reservoir, which is located at the intersection of a major fault and secondary fault zones. TKXX264 well has become a high-water-cut (nearly 90%) well due to the rise of bottom water during production. Therefore, to address high water cut in Well THXX264, polymer gel water shut-off has been implemented. Based on the geological development characteristics of this well, an injection volume of 140 m^3^ of URG has been designed. The injection pressure remains stable at approximately 15 MPa, with an hourly injection rate of 20–25 m^3^. After completing the operation in early April 2025 and shutting in the well for one week, the well is reopened for production. The URG can ensure a high strength grade and accumulate slowly until the bulk gel system is gelated completely and occupies the water flow fracture channel due to its viscoelastic properties and outstanding anti-dilution ability. The water cut of the well is reduced from 90% to 30%, and the stage cumulative production is 2416 t, as shown in Figure 15.

## 3. Conclusions

Based on the development and evaluation of URG, a study on the distribution patterns of URG in different fractured-vuggy reservoir structures was conducted using online NMR and core slice analysis. The conclusions formed are as follows:

(1) URG forms a dense 3D network structure with a minimum pore size of approximately 10 μm. The storage modulus (G′) and loss modulus (G″) of the gelated URG are 387.51 Pa and 131.48 Pa, respectively. The incorporated sulfonate functional groups enhance URG’s thermal stability and salt resistance.
(2) URG forms a gradient sealing zone with intensity decreasing from inlet to outlet in slab-fracture cores, showing effective plugging performance and strong erosion resistance during water flooding. The breakthrough pressure for subsequent water flooding in slab fractures core is 5.9 MPa.
(3) URG achieves uniform distribution in fractured-vuggy cores and migrates toward large vugs forming stable sealing segments during water flooding. The breakthrough pressure for subsequent water flooding in fractured-vuggy core is 8.1 MPa.
(4) URG forms stepwise distributed sealing layers in bead-like vug cores and enhances plugging efficiency through dynamic migration while exhibiting strong erosion resistance. The breakthrough pressure for subsequent water flooding in beaded-vug train core is 11.6 MPa.
(5) After the injection of 140 m^3^ URG for conformance control in THXX264 well, the water cut is reduced from 90% to 30%, and the cumulative oil increase in stage is 2416 t, which demonstrates remarkable water control effectiveness of URG.

## 4. Materials and Methods

### 4.1. Raw Materials Characteristics

Modified polyacrylamide JH140-1 (AM-co-AMPS, molecular weight 9 million Daltons, 25% hydrolysis), water soluble urea-formaldehyde resin crosslinker JD140-1, weak acid accelerator CJ140-1, thiourea stabilizer ZJ140-1, conditioning agent TJ140-1 and inhibitor KL140-1, were provided by Haipeng Chemical Technology Co., Ltd., Shanghai, China. Deuterium oxide (D_2_O, AR, 99.5%) were supplied by Shanghai Macklin Biochemical Technology Co., Ltd., Shanghai, China.

### 4.2. Experimental Instruments

SEM measurement Sigma 360, Carl Zeiss Co., Oberkochen, Germany. Rotary rheometer RS600, HAAKE, Karlsruhe, Germany. 80 V infrared spectrum analyzer, Bruker, Bremen, Germany. Raman Imaging Microscope DXR2xi, Thermo Scientific, Waltham, MA, USA. Core displacement equipment HW-II, Haian Co., Ltd., Nantong, China. Plunger pump 100DX (accuracy of velocity and pressure: ±0.5%), Teledyne Isco, Lincoln, NE, USA. NMR core displacement equipment MesoMR12-060H-I, Suzhou Niumag Analytical Instrument Co., Ltd., Suzhou, China.

### 4.3. Preparation and Analytical Method of URG

First of all, 0.1 wt% inhibitor KL140-1 and 0.6 wt% Modified polyacrylamide JH140-1 are slowly added into a beaker pre-filled with 100 mL salinity water (24 × 104 mg/L). Simultaneously, magnetic stirring is used to dissolve agents at a speed of 300 rpm until polymer particles are fully dispersed in the solution. To ensure polymer molecules dissolving fully in aqueous solutions, the resulting solution is aged at room temperature for 1 h after stirring after 45 min. Then, restart magnetic stirring and keep it speed at 200 rpm, 0.5 wt% JD140-1, 0.6 wt% CJ140-1, 0.5 wt% TJ140-1 and 0.3 wt% ZJ140-1 are added to the polymer solution in turn. The URG solution is prepared after 10 min [19].

URG was investigated using SEM, rheological analysis, FTIR, and Raman spectroscopy. Leveraging 3D printing technology, typical structural models of fractured-vuggy reservoirs were fabricated. The distribution patterns of the URG within different fractured-vuggy configurations were analyzed online NMR technology and core slice.

### 4.4. Design and Manufacture of Fractured-Vuggy Simulation Models

**Figure 16:**
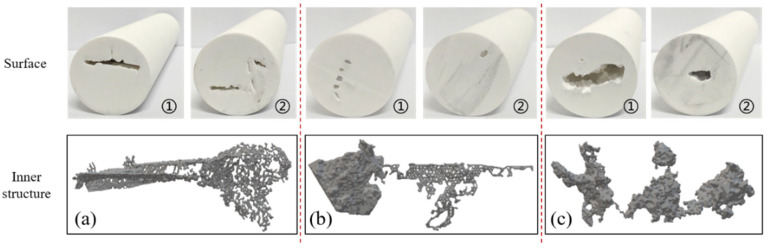
Physical specimen of 3D printed core and its internal fractured-vuggy network: (**a**) Slab fractures model (**b**) Fractured-vuggy model (**c**) Beaded-vug train model.

To achieve the highest fidelity in reconstructing the true internal architecture of fractured-vuggy carbonate reservoirs, 3D printing technology was employed to fabricate three different types of typical fractured-vuggy core structures in this study. Calcium carbonate powder, aluminum oxide, and zirconium oxide were mixed in a specific ratio (6:2:2); the simulation model parameters were designed according to similarity criterion, which includes geometric similarity, flow similarity, and dynamic similarity in fractured-vuggy reservoirs [38]. The matrix permeability of physical model is less than 0.01 mD and the size of the physical model is ϕ25 mm × 100 mm. Reservoir types can be divided into slab fracture system, fractured-vuggy structure and aligned vug cluster. Slab fractures model has a long extension with uniform aperture. The fractured-vuggy model mainly developed a fracture dissolution zone. Beaded-vug train model typically demonstrated anisotropic connectivity behavior and was controlled by tectonic fractures. Different inner structures of theoretical models and designed physical models are shown in Figure 16.

### 4.5. Experimental Process

#### 4.5.1. Conventional Experimental Methodology

**Figure 17:**
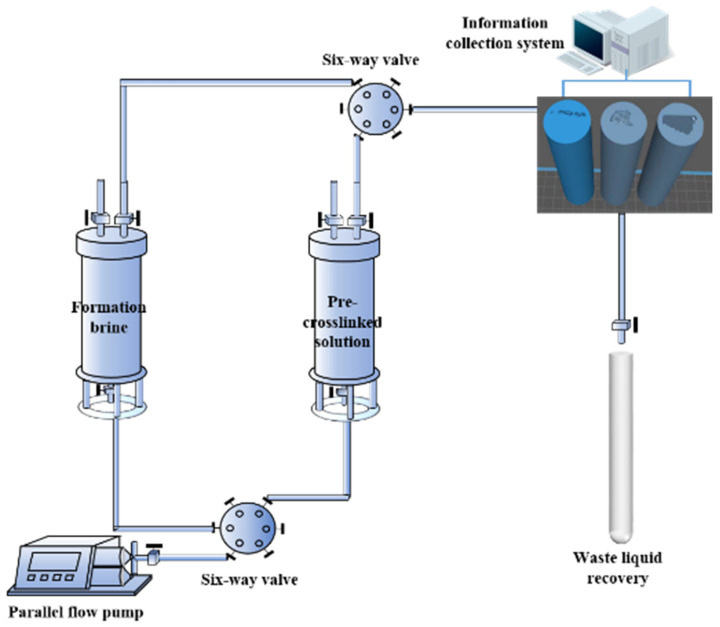
Schematic diagram of the physical simulation experiment.

(1) Vacuum the model for 6 h. Saturate water and record water saturation. Then, put these simulation models in high temperature (140 °C) oven for 24 h.
(2) Primary water flooding is carried out at 0.5 mL/min until initial water breakthrough. Then, gel solution is injected 0.3 PV into the simulation model at 0.1 mL/min. The specific injection flow rates selected for the laboratory experiments are determined based on relevant literature from previous similar plugging tests, with the requirement to ensure that pressure during gel injection gradually increases. In order to prevent bulk gel plugging the pipeline, a small amount of water is injected to clean the pipeline after finishing the gel solution injection. The physical model is aged at high temperature for 5 days until the gel solution is gelatinized.
(3) Subsequent water flooding is injected at 0.5 mL/min after bulk gel system gelated completely. The subsequent water flooding is stopped when the water is observed at the outlet. The schematic diagram of the physical simulation experiment is shown in Figure 17.

#### 4.5.2. Online NMR Experimental Methodology

Repeat conventional experimental methodology step 1 to step 3. Deuterium oxide (D_2_O, without NMR signals) is employed as the experimental displacing fluid, which constitutes the primary distinction. Meanwhile, NMR T_2_ spectrum analysis is performed during core displacement experiments [39].

## Data Availability

The original contributions presented in this study are included in the article. Further inquiries can be directed to the corresponding author(s).
